# Frequent use of hospital inpatient services during a nine year period: a retrospective cohort study

**DOI:** 10.1186/s12913-017-2285-1

**Published:** 2017-05-12

**Authors:** Adelle M. Springer, John R. Condon, Shu Q. Li, Steven L. Guthridge

**Affiliations:** 1 Health Gains Planning Branch, Northern Territory Department of Health, PO Box 40596, Casuarina, NT 0811 Australia; 2 Menzies School of Health Research, Charles Darwin University, PO Box 41096, Casuarina, NT 0811 Australia

**Keywords:** Frequent use, Hospital, Admissions, Inpatient, Aboriginal, Non-Aboriginal, Northern Territory, Cohort

## Abstract

**Background:**

Frequent use (FU) of hospital services impacts on patients and health service expenditure. Studies examining FU in emergency departments and inpatient settings have found heterogeneity and the need to differentiate between potentially preventable FU and that associated with ongoing management of complex conditions. Psychosocial factors have often been reported as underpinning or exacerbating the phenomena.

Most FU studies have been limited by time, to a single study site, or restricted to specific diagnoses or patient groups. This study provides a comprehensive description of adult patient characteristics, conditions and risk factors associated with FU, based on admissions to the five public hospitals in the Northern Territory (NT) of Australia over a nine year period. The study population is distinctive comprising both Aboriginal and non-Aboriginal patients.

**Methods:**

Data on all inpatient episodes in NT public hospitals between 2005 and 2013 was analysed to identify patients with any FU (four or more episodes within any 12-month period) and measure FU duration (number of FU years) and intensity (mean number of episodes per FU year). Pregnancy, alcohol-related and mental health condition flags were assigned to patients with any episode with relevant diagnoses during the study period. Multivariate analysis was used to assess factors associated with any FU, FU duration and FU intensity, separately for Aboriginal and non-Aboriginal patients.

**Results:**

Of people with any inpatient episodes during the study period, 13.6% were frequent users (Aboriginal 22%, non-Aboriginal 10%) accounting for 46.6% of all episodes. 73% of frequent users had only one FU year. Any FU and increased FU duration were more common among individuals who were: Aboriginal; older; female; and those with a pregnancy, alcohol or mental health flag. Having two or more alcohol-related episodes in the nine-year period was strongly associated with any FU for both Aboriginal (odds ratio 8.9, 95% CI. 8.20–9.66) and non-Aboriginal patients (11.5, 9.92–13.26).

**Conclusion:**

For many people, frequent inpatient treatment is necessary and unavoidable. This study suggests that damage arising from excessive alcohol consumption (either personal or by others) is the single most avoidable factor associated with FU, particularly for Aboriginal people.

**Electronic supplementary material:**

The online version of this article (doi:10.1186/s12913-017-2285-1) contains supplementary material, which is available to authorized users.

## Background

Health system providers in many countries have been challenged by the disproportionate demand on services by ‘frequent user’ patients, which impacts on the wellbeing of those patients, resources available for other patients and health service expenditure. Previous studies have examined frequent emergency department (ED) attendances [1–4] and frequent hospital admissions [5–7]. While the occurrence of frequent use (FU) has been widely reported, the definition has varied. Four visits in 12 months is the typical threshold in ED studies, but three, five and ten visits have also been used [1, 2, 4]. Studies of frequent admissions commonly use three admissions in a 12 month period, although some have used four admissions or have varying time periods [6, 7].

However, there is some consensus that frequent users are not homogenous and that clusters of subgroups exist, the reported characteristics of which have varied in acuity diagnostic-related group and payment method, but also across ethnicity, socio-demographic factors, and history of violence or substance abuse [3, 6]. For some, frequent hospital admissions may be mitigated by increased access to primary care, earlier intervention [8] or tailored case management [9].

There is evidence that the interplay of mental health, alcohol and socio-economic factors (inadequate housing, deprivation and poor social networks) acts to underpin or exacerbate FU [4, 7, 10–12]. Excessive alcohol consumption is a well-documented problem in the Northern Territory (NT) of Australia [13], where for 2014 consumption was estimated to be 12.2 litres per person [14], compared with the national estimate of 9.7 litres available for consumption per person [15].

This study examined patient characteristics and factors associated with FU of hospital inpatient services including alcohol and mental health in the NT, a large and remote jurisdiction in northern and central Australia where Aboriginal people comprise 30% of the population (compared with 3% nationally) [16]. The Aboriginal Australian population (Aboriginal and Torres Strait Islander peoples) has much worse social, economic and environmental conditions, and a much greater burden of chronic disease, associated risk factors and comorbidities than Australians generally, including pervasive end-stage kidney disease, and greater use of acute care services [17–19]. Excessive alcohol consumption is also much more common among Aboriginal than other Australians; one study estimated that in the period from 1998–99 to 2008–09 the annual alcohol-attributable hospitalisation rate for the NT Aboriginal population was 329.2 per 10,000, which was nearly seven times the corresponding rate for the non-Aboriginal population (49.6 per 10,000) [20].

The NT has five public hospitals located in the NT’s five regional cities/towns ranging in size from the Royal Darwin Hospital, a 343 bed teaching hospital serving a population of 140,000, to the Gove District Hospital, a 30 bed district hospital serving the isolated mining town of Nhulunbuy and remote Aboriginal communities in Arnhem Land. All five hospitals are the primary hospitals for their region, and two are also referral hospitals for the three small district hospitals; patients may be serviced by more than one hospital. The NT has had a unique health client identifier since 1991 that enables linkage of all hospital episodes of care for each person in the five NT public hospitals. This study was therefore able to investigate FU duration and intensity by Aboriginal and non-Aboriginal patients, across multiple sites and over a nine year period 2005–2013. To the best of our knowledge, this study is therefore unmatched in its extended study period and comprehensive coverage of the client adult population and its frequent use of inpatient services.

## Methods

Data on all inpatient episodes in the five NT public hospitals from 2005 to 2014 was extracted from the NT Hospital Separations Dataset. This dataset contains a summary of each inpatient episode, including the NT health unique client identifier (the hospital registration number) that allows linkage of all episodes for each patient in any of the five hospitals.

The study included adults (15 years and over) and the variables were: age at first episode in the study period; sex; Indigenous status; residence (urban/remote) at first episode; date of episode; primary and 19 secondary diagnosis codes; and mode of separation. Diagnoses are coded at time of hospital separation by qualified coders using the International Classification of Diseases version 10, Australian modification (ICD-10-AM). Demographic information in NT hospital data has a high level of accuracy, with Indigenous status estimated to have 98% consistency between electronic patient records and self-report at interview [21]. Data from the one private hospital in the NT was not included.

**Fig. 1:**
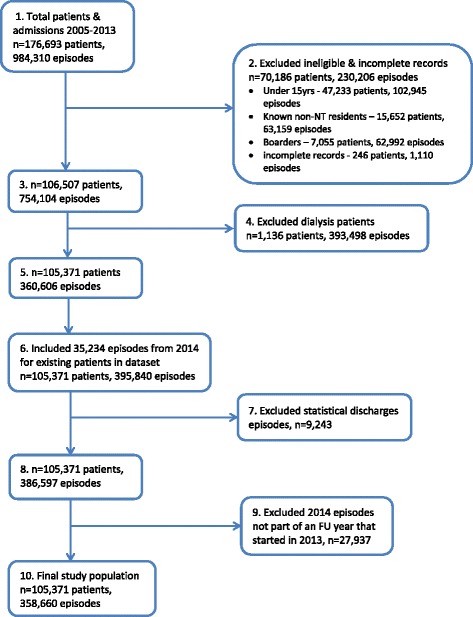
Data reduction flow diagram

From an initial 176 693 patients who had 984 310 episodes between 2005 and 2013 (Fig. 1), 69 940 ineligible patients with 229 096 episodes were excluded. Ineligible patients were interstate or overseas residents; children (under 15 years), who were considered likely to have significantly different characteristics and risk factors for FU; or episodes recorded as ‘boarder’ episodes (e.g., the mother of a sick child). A further 246 patients and their 1 110 episodes were also excluded because records were incomplete (e.g., missing key demographic data such as Indigenous status, age or date of birth, or missing the primary diagnosis).

In the NT renal dialysis is categorised as an inpatient episode. This was a priori acknowledged as such a dominant reason for frequent admissions in the NT that 1 136 patients with any episode during the study period with a primary diagnosis of dialysis for chronic kidney disease (393 498 episodes) were excluded from the analysis.

A ‘statistical discharge’ is an administrative reclassification of a continuing inpatient episode during which, without leaving the hospital, a patient is ‘statistically discharged’ and ‘statistically readmitted’ on the same day. Episodes ending in a statistical discharge were consolidated into a single episode. For hospital episodes involving statistical discharges, the diagnoses recorded for the first episode in each statistical discharge/episode series were used for analysis except when the first episode was an emergency department episode, in which case diagnoses for the second episode (in a hospital ward) were used.

A frequent user was defined as a patient who had four or more admissions in any 365 day period commencing on or after 1 January 2005. A subsequent FU year could not commence until day 366 after the admission date of the first episode in the previous FU year. Admissions for FU years that commenced in 2013 and ended in 2014 were included; admissions in FU years that commenced in 2004 and ended in 2005 were not included.

FU duration was defined as the number of FU years, to a maximum of nine, during the study period. FU intensity was defined as the average number of episodes per FU year for each frequent user; episodes and person-time outside each patient’s FU years were not included. Intensity was categorised as: ‘often’, an average of four to less than five episodes per FU year; ‘high’, five to less than eight; and‘very high’, eight or more episodes. These cut points were chosen to include approximately half of frequent users in the bottom category and 10% in the top category (actual proportions were 47.1 and 11.7% respectively).

An indicator flag for alcohol-related risk was applied to patients if any of their episodes had a primary diagnosis code that was wholly or partially attributable to alcohol; i.e., had an alcohol population aetiologic fraction of 40% or greater or, in the case of injury or poisoning, any alcohol-related external cause code (see Additional file 1: Tables S1 and S2) [20, 22, 23]. Similarly an indicator flag for mental health related risk was applied to patients if any episode during the study period had a primary diagnosis code of a ‘mental and behavioural disorder’ (ICD-10-AM Chapter 5). Those with any primary diagnosis under Chapter 15 *‘pregnancy, childbirth and the puerperium’* were flagged as having a pregnancy-related condition.

Univariate analysis compared demographic and clinical characteristics of frequent and occasional users; and compared FU intensity and duration for frequent users. Univariate analysis was stratified by Aboriginal status and number of FU years (categorized as 0/1/2+) at the person level (frequency proportions; median age; and means for number of FU years per person, episodes per FU year, and length of stay per episode) and at the episode level (frequency proportions).

Multivariable analysis using logistic regression was used to assess associations between the outcomes ‘any FU’ and ‘FU duration’ (single versus multiple FU years) and explanatory variables: Indigenous status; age at first episode; sex; urban/remote residence at first episode; a mental health flag; an alcohol-related flag; and a pregnancy flag during the study period. Associations between these same explanatory variables and the outcome of ‘FU intensity’ (average number of inpatient episodes per FU year) were assessed using a generalised estimating equation model, which adjusted for the within subject correlation and the parametric distribution of episodes [24].

Multivariable analysis was done separately for Aboriginal and non-Aboriginal patients as the effect of most factors associated with FU was found to be different for each group. Stata Version 14 was used for statistical analysis.

The study was approved by the Human Research Ethics Committee of the Northern Territory Department of Health and the Menzies School of Health Research (HREC 2013–2067).

## Results

The study population comprised 105 371 eligible patients with 358 660 inpatient episodes (including 6 413 episodes in 2014 that were part of a FU year commencing in 2013), between 2005 and 2013. Of these, 32 423 (30.8%) were Aboriginal people, who had 159 496 (44.5%) episodes and 72 948 were non-Aboriginal people who had 199 164 episodes (Table 1). Those who had ever been a frequent user were 13.6% of the study population and accounted for 46.6% of episodes.

**Table 1:** NT public hospital episodes and inpatient characteristics, by Aboriginal status, 2005–2013

	Aboriginal		Non-Aboriginal		TOTAL	
	Patients	Episodes	Patients	Episodes	Patients	Episodes
Total study population (n)	32 423	159 496	72 948	199 164	105 371	358 660
Female (%)	58.2	62.1	52.5	51.9	54.3	56.5
Median age (years)^a^	31		39		36	
Rural/remote residence (%)^b^	69.1	66.7	18.6	17.1	34.2	39.2
Alcohol-related flag (%)	24.4	13.3	4.6	2.8	10.7	7.5
Mental health flag (%)	9.4	4.7	4.4	3.2	6.0	3.9
Pregnancy-related flag (%)	26.3	15.3	21.7	16.5	23.1	16.0
Any frequent use (FU) (%)	21.7	57.8	10.0	37.7	13.6	46.6
1 FU year (%)	14.3	23.9	8.0	22.3	9.9	23.0
2+ FU years (%)	7.4	33.9	2.0	15.4	3.7	23.6

^a^Based on age at first episode

^b^Based on residence at first episode - excludes 70 (0.22%) Aboriginal persons with 102 (0.06%) episodes and 36 (0.05%) non-Aboriginal persons with 57 (0.03%) episodes with recorded unknown residence

Any FU was twice as common for Aboriginal patients (21.7%) than non-Aboriginal patients (10.0%) (Table 1), with a crude odds ratio of 2.50 (95% CI. 2.41–2.59). Most frequent users had a single FU year, although increased FU duration was higher for Aboriginal patients; crude odds ratio of 2.02 (95% CI. 1.88, 2.18).

Both Aboriginal and non-Aboriginal frequent users were older than occasional users and more likely to have a risk flag for a mental health or alcohol-related condition (Table 2). Around one third of all frequent users in both subgroups were pregnant women, the majority of whom had just a single FU year.

**Table 2:** NT public hospital inpatient characteristics (persons and episodes), by Aboriginal status and frequent use duration, 2005–2013

	Aboriginal patients			Non-Aboriginal patients		
	occasional	1 FU yr	2+ FU yrs	occasional	1 FU yr	2+ FU yrs
Persons^a^						
Total number	25 379	4 644	2 400	65 650	5 814	1 484
Female (%)	56.4	65.4	63.7	52.5	55.6	41.0
Median age (years)^b^	30	33	36	38	45	55
Rural/remote residence^c^ (%)	69.6	68.3	65.6	18.9	16.5	14.8
Alcohol-related flag (%)	18.5	36.8	61.9	3.9	9.8	18.8
Mental health flag (%)	6.4	13.8	33.2	3.7	9.3	18.5
Pregnancy-related flag (%)	25.3	33.5	23.6	21.1	30.6	14.4
Frequent use (FU) years						
Number of FU years		4 644	7 207		5 814	3 835
Mean FU years per person		1	3.0		1	2.6
(95% C.I.)			(2.9, 3.1)			(2.5, 2.6)
Number of episodes in FU years		23 853	45 521		33 171	26 426
Mean episodes per FU year		5.1	6.3		5.7	6.9
(95% C.I.)		(5.1, 5.2)	(6.2, 6.4)		(5.6, 5.8)	(6.7, 7.1)
Number of inpatient days in FU years		116 499	209 397		138 230	115 161
Mean LOS (days) per episode		4.88	4.60		4.17	4.36
Episodes^d^						
Total number	67 358	38 096	54 042	124 211	44 342	30 611
Female (%)	61.7	65.5	60.3	54.4	53.1	40.1
Rural/remote residence^c^ (%)	70.1	67.7	61.8	18.5	16.0	13.4
Alcohol-related condition (%)	10.5	11.8	18.0	2.5	3.0	3.6
Mental health condition (%)	3.7	4.1	6.4	2.7	3.3	4.9
Pregnancy-related condition (%)	20.5	18.2	6.7	18.9	17.3	5.8

^a^number of persons

^b^Based on age at first episode

^c^Based on residence at first episode - excludes records with unknown residence as noted in Table 1

^d^number of inpatient episodes

FU intensity was similar for Aboriginal and non-Aboriginal patients and for those with single or multiple FU years, with mean number of episodes between 5.1 and 6.9 episodes per FU year.

Risk flags for alcohol-related and mental health conditions were much more prevalent among Aboriginal than non-Aboriginal patients, for both occasional and frequent users (Table 2). Among Aboriginal patients with 2+ FU years the prevalence of alcohol-related condition risk flags (61.9%) was over three times that of their non-Aboriginal counterparts (18.8%). Alcohol-related condition flags were 15.4% of total Aboriginal FU episodes. Having a risk flag for a mental health condition was one and a half to nearly twice as prevalent for Aboriginal than for non-Aboriginal FU patients.

The most common reasons (at ICD Chapter level) for Aboriginal people’s frequent use were respiratory diseases and injury and poisoning (Table 3). Pneumonia, bronchitis, bronchiectasis and chronic obstructive pulmonary disease were the most common primary diagnoses among the respiratory diseases. Most common ICD Chapters were broader for non-Aboriginal frequent users, although ‘Factors influencing health status & contact with health services’ dominated in the very high intensity categories (Table 3); this chapter includes chemotherapy for cancer, the most common primary diagnosis for very high intensity FU episodes.

**Table 3:** Leading ICD Chapters for Aboriginal and non-Aboriginal episodes, by frequent use intensity and duration, 2005–2013

Aboriginal		Non-Aboriginal	
Single FU year			
v high users^a^ (%) *n* = 4 871		(%) *n* = 10 577	
21.2	health status & health services factors	38.4	health status & health services factors
12.2	pregnancy-related conditions	10.0	neoplasms
10.3	injury, poisoning & external causes	9.2	pregnancy-related conditions
high users^b^ (%) *n* = 15 592		(%) *n* = 16 067	
19.0	pregnancy-related conditions	20.2	pregnancy-related conditions
16.4	injury, poisoning & external causes	11.0	health status & health services factors
8.9	respiratory diseases	8.8	digestive system diseases
often users^c^ (%) *n* = 17 633		(%) *n* = 17 698	
19.1	pregnancy-related conditions	19.6	pregnancy-related conditions
18.3	injury, poisoning & external causes	11.4	diseases of the digestive system
8.8	respiratory diseases	9.4	injury, poisoning & external causes
Multiple FU years			
v high users (%) *n* = 12 216		(%) *n* = 10 973	
19.9	respiratory diseases	35.2	health status & health services factors
13.6	symptoms, & abnormal findings, not elsewhere classified	11.7	endocrine diseases
10.6	injury, poisoning & external causes	8.2	blood & blood forming organs diseases
high users (%) *n* = 28 009		(%) *n* = 13 025	
18.2	injury, poisoning & external causes	11.0	symptoms & abnormal findings, not elsewhere classified
14.4	respiratory diseases	10.0	circulatory system diseases
9.6	Symptoms & abnormal findings, not elsewhere classified	10.0	respiratory diseases
often users (%) *n* = 12 216		(%) *n* = 6 613	
18.5	injury, poisoning & external causes	11.3	respiratory diseases
11.8	respiratory diseases	10.9	symptoms & abnormal findings, not elsewhere classified
10.2	pregnancy-related conditions	10.5	circulatory system diseases

^a^very high users - average 8+ episodes per FU year

^b^high users - average 5.0–7.9 episodes per FU year

^c^often users - average 4.0–4.9 episodes per FU year

Pregnancy was common for both Aboriginal and non-Aboriginal patients with a single FU year, but among those with multiple FU years it was only a top three ICD-10 category for Aboriginal ‘Often’ users.

In multivariable analysis, having any FU year was more common in older age-groups and a little more common for urban than rural residents (Table 4). Any FU year was more common for females among Aboriginal patients, but more common for males among non-Aboriginal patients. Odds of having any FU year were higher among those who had a pregnancy during the study period, a risk flag for a mental health condition or single alcohol-related condition. Having had two or more episodes for alcohol-related conditions was strongly associated with any FU, more so for non-Aboriginal patients (OR 11.47, 95% CI. 9.92, 13.26) than Aboriginal patients (OR 8.90, 95% CI. 8.20, 9.66).

**Table 4:** Multivariate analysis of adult inpatient frequent use, stratified by Aboriginal status, NT public hospitals, 2005–2013

	Aboriginal		Non-Aboriginal	
	odds ratio	95% CI.	odds ratio	95% CI.
Any frequent use year				
Female (c/w male)	1.39	1.30, 1.49	0.84	0.79, 0.90
Age groups (c/w 15–29 years)^a^				
30–44 years	1.75	1.63, 1.89	0.92	0.85, 0.99
45–60 years	3.01	2.73, 3.31	2.24	2.05, 2.44
60+ years	3.98	3.55, 4.47	5.15	4.74, 5.60
Rural/remote (c/w urban)^b^	0.91	0.85, 0.96	0.85	0.80, 0.91
Alcohol-related (AR) condition (c/w 0)				
1 AR episode	1.75	1.60, 1.91	1.78	1.56, 2.02
2+ AR episode	8.90	8.20, 9.66	11.47	9.92, 13.26
Mental health flag	2.80	2.56, 3.07	3.50	3.18, 3.85
Pregnancy related flag	2.42	2.22, 2.64	4.02	3.69, 4.38
Increased frequent use duration among frequent users^c^				
Female (c/w male)	1.32	1.17 1.49	0.86	0.75, 0.99
Age groups (c/w 15–29 years)^a^				
30–44 years	1.46	1.27, 1.68	0.87	0.70, 1.08
45–60 years	1.60	1.34, 1.90	1.33	1.06, 1.67
60+ years	1.36	1.09, 1.70	1.82	1.46, 2.27
Rural/remote (c/w urban)^b^	0.94	0.84, 1.05	0.82	0.69, 0.96
Alcohol-related (AR) condition (c/w 0)				
1 AR episode	1.32	1.12, 1.55	1.11	0.86, 1.44
2+ AR episode	2.88	2.55, 3.26	2.21	1.80, 2.72
Mental health flag	2.26	1.98, 2.57	1.94	1.63, 2.31
Pregnancy related flag	0.83	0.71, 0.98	0.70	0.55, 0.89

^a^Based on age at first episode

^b^Based on residence at first episode

^c^Two or more compared with one frequent use year

For FU duration, having two or more FU years was more common for older age-groups, for females (Aboriginal only), those who had a mental health flag and those who had two or more episodes for alcohol-related conditions, but odds ratios were mostly smaller than for associations with any FU year (Table 4). Two or more FU years was less common for women who had a pregnancy during the study period.

For FU intensity, the number of inpatient episodes per FU year was associated with several factors but the effect size (the greater or fewer number of episodes) was small and of little practical consequence for all associations; eg. Aboriginal frequent users had 0.12 fewer episodes (95% CI. -0.18, −0.07) per FU year than non-Aboriginal frequent users (Additional file 1: Table S3).

## Discussion

Frequent users are a small proportion of inpatients in NT public hospitals but account for a substantial proportion of episodes. The overall proportions in the NT echo those found in a UK study of older patients [5], while in the NT these are clearly driven by FU by Aboriginal more than non-Aboriginal people.

The nature of frequent use is very different for Aboriginal and non-Aboriginal patients. The prevalence of any FU by Aboriginal patients was double and prevalence of multiple FU years over three times that of non-Aboriginal patients. Aboriginal FUs are younger and more commonly female that non-Aboriginal frequent users. As it is currently categorised in the NT, kidney disease singularly dominates hospital admissions and is a major burden for the health system and the Aboriginal population, who experience increased risk of comorbidities and mortality [17, 19]. In this study, which excluded patients with a history of renal dialysis, the most common reason for FU was chemotherapy for cancer for non-Aboriginal people, while injury and respiratory diseases were among the leading drivers of Aboriginal FU. Injury was one of the top three reasons for FU episodes at all levels of intensity and duration for Aboriginal frequent users, accounting for 10–18% of FU episodes, compared to 9% at most for non-Aboriginal frequent users. Alcohol was the largest risk factor for FU among those variables tested. Aboriginal people suffer higher alcohol-attributable morbidity and mortality [13, 20] and the higher prevalence of alcohol-related conditions among Aboriginal patients clearly contributed to their excess inpatient FU. 62% of Aboriginal multi-year frequent users had an alcohol risk flag but only 18% of their inpatient episodes were directly attributable to alcohol, indicating that their alcohol risk contributed to other health problems.

A risk flag for an alcohol-related condition is not restricted to the patient’s own involvement with alcohol; instead they may be a victim or recipient of an adverse outcome due to another’s alcohol consumption. Assault is an obvious example, which may be reflected in the prominence of injury as a reason for FU by Aboriginal people. This may be particularly the case for Aboriginal women; only 18% of NT Aboriginal adult women report risky/high risk alcohol consumption compared to 34% of men, [24] but women account for 64% of Aboriginal (compared to 39% of non-Aboriginal) high-duration frequent users.

Psycho-social issues regularly feature in FU studies and (as highlighted here) the concomitant presence of both alcohol-related and mental health conditions is not uncommon [1, 5, 10, 11]. Studies have also demonstrated that risk factors for FU of hospital services include these psycho-social stressors along with chronic disease, poverty, inadequate housing, social marginalisation and low socio-economic status [4, 7, 11, 12, 25]. Such health and environmental factors may contribute to increased FU in the NT, particularly (but not only) by Aboriginal patients.

To the best of our knowledge this study is innovative in presenting the picture of FU over nearly a decade. While there is attrition after the first FU year as found in some ED studies [1, 26], there is a smaller but substantial subset of the FU population who experience protracted periods of frequent use.

There are limitations to the study. Our assumption of a stable NT population over the nine years is appropriate for the Aboriginal population given its low level of migration into and out of the NT [27], but it is more of a limitation for the non-Aboriginal population that has high interstate mobility [28]. Additionally, data were not available for the one private hospital in the NT. These factors may result in an underestimation of frequent use among non-Aboriginal patients as some who were frequent users but moved interstate, or were frequent users in the private hospital would have been missed in this study.

## Conclusion

FU is more common in Aboriginal people. The strongest risk factor identified in this study is a history of damage attributable directly or indirectly to excessive alcohol consumption, particularly for Aboriginal people. This is a major social problem in the NT for both Aboriginal and non-Aboriginal people and one that is not within the control of acute care services.

There are some conditions for which FU is inevitable and others for which integrated programs may moderate excessive and avoidable admissions leading to better health outcomes and reduced costs [8, 9]. Respiratory disease as a prominent feature of FU at all levels and duration among the Aboriginal population is indicative of the social deprivation and disparity that persists.

Reducing the contribution of alcohol to FU however requires change at individual, community and society levels. Education, legislation and policies that help minimise the proportion of people exposed to the risk of excessive alcohol consumption will affect a decrease in the overall FU of hospital services in the NT.

## Additional file

Additional file 1: Table S1. Wholly and partially alcohol-attributable conditions for hospital admissions with alcohol population aetiologic fractions of 100% and ≥40%. **Table S2.** ICD-10-AM Chapter as abbreviated in Table 3. **Table S3.** General estimating equation analysis of inpatient frequent use intensity*, NT public hospitals, 2005-2013. (DOCX 18 kb)

## Abbreviations

95% CI.: 95% confidence interval
ABS: Australian Bureau of Statistics
ED: Emergency Department
FU: Frequent use
ICD-10-AM: International classification of diseases and related health problems, 10th revision, Australian modification
NT: Northern Territory
OR: Odds ratio
