# Supplementary material for: Frequent use of hospital inpatient services during a nine year period: a retrospective cohort study
Source: BMC Health Serv Res. 2017 May 12;17:348. doi: 10.1186/s12913-017-2285-1 (PMC5427599; doi:10.1186/s12913-017-2285-1)
Supplement: Additional file 1: Table S1. — Wholly and partially alcohol-attributable conditions for hospital admissions with alcohol population aetiologic fractions of 100% and ≥40%. Table S2. ICD-10-AM Chapter as abbreviated in Table 3. Table S3. General estimating equation analysis of inpatient frequent use intensity*, NT public hospitals, 2005-2013. (DOCX 18 kb) [file 12913_2017_2285_MOESM1_ESM.docx]

Additional files

Table S1 Wholly and partially alcohol-attributable conditions for hospital admissions with alcohol population aetiologic fractions of 100% and >40%

| **ICD-10-AM code (100%)** | **ICD-10-AM description** |
| --- | --- |
| F10.0- F10.9 | mental and behavioural disorders due to alcohol use |
| I42.6 | alcoholic cardiomyopathy |
| G31.2 | degeneration of nervous system due to alcohol |
| G62.1 | alcoholic polyneuropathy |
| G72.1 | alcoholic myopathy |
| K29.2 | alcoholic gastritis |
| K70 | alcoholic liver disease |
| K85.2 | alcohol-induced acute pancreatitis |
| K86.0 | alcohol-induced chronic pancreatitis |
| T51.0 | toxic effects of alcohol - ethanol |
| T51.1 | toxic effects of alcohol - methanol |
| T51.9 | toxic effects of alcohol – alcohol unspecified |
| X45 | accidental poisoning by and exposure to alcohol |
| Y15 | poisoning by and exposure to alcohol, undetermined intent |
| **ICD-10-AM code (>40%)** |  |
| C01-C06, C09- C10, C12 - C14 | oropharyngeal cancer |
| C15 | oesophageal cancer |
| C22 | liver cancer |
| C32 | laryngeal cancer |
| G40, G41 | epilepsy & status epilepticus |
| I85, I98.2, 198.20, I98.21 | oesophageal varices |
| K22.6 | gastro oesophageal haemorrhage |
| K74.3 - K74.6, K76.0, K76.9 | unspecified liver cirrhosis |
| X85-Y09, Y87.1 | assault |
| K85.9, K86.0, K85.2 | acute pancreatitis, unspecified [collectively] |
| X65 | intentional self-poisoning by and exposure to alcohol |

**Table** S2 **ICD-10-AM Chapter as abbreviated in Table 3**

| **Table term** | **ICD Chapter number & name** |
| --- | --- |
| Neoplasms | 2 Neoplasms |
| Blood & blood forming organs diseases | 3 Diseases of the blood & blood forming organs & certain disorders involving the immune mechanism |
| Endocrine diseases | 4 Endocrine, nutritional & metabolic diseases |
| Circulatory system diseases | 9 Diseases of the circulatory system |
| Respiratory diseases | 10 Diseases of the respiratory system |
| Digestive system diseases | 11 Diseases of the digestive system |
| Pregnancy-related conditions | 15 Pregnancy, childbirth and the puerperium |
| Symptoms, & abnormal findings, not elsewhere classified | 18 Symptoms, signs & abnormal clinical & lab findings, not elsewhere classified |
| Injury, poisoning & external causes | 19 Injury, poisoning & certain other consequences of external causes |
| Health status & health services factors | 21 Factors influencing health status & contact with health services |

**Table** S3 **General estimating equation analysis of inpatient frequent use intensity*, NT public hospitals, 2005-2013**

|  | **Number of episodes** | **95% CI** |
| --- | --- | --- |
| Aboriginal [c/w Non-Aboriginal] | -0.12 | -0.18, -0.07 |
| female [c/w male] | -0.01 | -0.05, 0.03 |
| age [per single year of age]^**^ | -0.00001 | -0.00002, 0.00001 |
| rural/remote [c/w urban]^†^ | -0.05 | -0.10, -0.01 |
| alcohol-attributable condition | 0.01 | -0.03, 0.05 |
| mental health condition |  |  |
| Non-Aboriginal | -0.12 | -0.20, -0.04 |
| Aboriginal | 0.09 | 0.02, 0.15 |
| Pregnancy-related condition | -0.09 | -0.14, -0.05 |
| Non-Aboriginal | -0.21 | -0.28, -0.14 |
| Aboriginal | -0.10 | -0.16, -0.04 |

* Number of episodes per FU year

^88^ Based on age at first episode, squared

† Based on residence at first episode
